# Seasonality and shift in age-specific malaria prevalence and incidence in Binko and Carrière villages close to the lake in Selingué, Mali

**DOI:** 10.1186/s12936-016-1251-4

**Published:** 2016-04-18

**Authors:** Mahamoudou Touré, Daouda Sanogo, Soumaila Dembele, Sory Ibrahima Diawara, Karen Oppfeldt, Karin L. Schiøler, Dade Ben Haidara, Sékou F. Traoré, Michael Alifrangis, Flemming Konradsen, Seydou Doumbia

**Affiliations:** Malaria Research and Training Centre-Faculty of Medicine and Dentistry, University of Sciences, Techniques and Technologies of Bamako (USTTB), Bamako, BP 1805 Mali; Global Health Section, Department of Public Health, Faculty of Health and Medical Sciences, University of Copenhagen, Øster Farimagsgade 5, P.O. Box 2099, 1014 Copenhagen, Denmark; Centre for Medical Parasitology, Department of International Health, Immunology and Microbiology, Faculty of Health and Medical Sciences, University of Copenhagen, Copenhagen, Denmark; Department of Clinical Microbiology and Department of Infectious Diseases, Copenhagen University Hospital (Rigshospitalet), Øster Farimagsgade 5, Building 22-23, 1014 Copenhagen, Denmark; BØRNEfonden, OttoMonsteds Gade 5, Copenhagen, Denmark

**Keywords:** Malaria parasitaemia, Malaria incidence, Seasonality, Agro-hydropower, Selingué, Mali

## Abstract

**Background:**

Malaria transmission in Mali is seasonal and peaks at the end of the rainy season in October. This study assessed the seasonal variations in the epidemiology of malaria among children under 10 years of age living in two villages in Selingué: Carrière, located along the Sankarani River but distant from the hydroelectric dam, and Binko, near irrigated rice fields, close to the dam. The aim of this study was to provide baseline data, seasonal pattern and age distribution of malaria incidence in two sites situated close to a lake in Selingué.

**Methods:**

Geographically, Selingué area is located in the basin of Sakanrani and belongs to the district of Yanfolila in the third administrative region of Mali, Sikasso. Two cross-sectional surveys were conducted in October 2010 (end of transmission season) and in July 2011 (beginning of transmission season) to determine the point prevalence of asymptomatic parasitaemia, and anaemia among the children. Cumulative incidence of malaria per month was determined in a cohort of 549 children through active and passive case detection from November 2010 through October 2011. The number of clinical episodes per year was determined among the children in the cohort. Logistic regression was used to determine risk factors for malaria.

**Results:**

The prevalence of malaria parasitaemia varied significantly between villages with a strong seasonality in Carrière (52.0–18.9 % in October 2010 and July 2011, respectively) compared with Binko (29.8–23.8 % in October 2010 and July 2011, respectively). Children 6–9 years old were at least twice more likely to carry parasites than children up to 5 years old. For malaria incidence, 64.8–71.9 % of all children experienced at least one episode of clinical malaria in Binko and Carrière, respectively. The peak incidence was observed between August and October (end of the rainy season), but the incidence remained high until December. Surprisingly, the risk of clinical malaria was two- to nine-fold higher among children 5–9 years old compared to younger children.

**Conclusions:**

A shift in the peak of clinical episodes from children under 5–9 years of age calls for expanding control interventions, such as seasonal malaria chemoprophylaxis targeting the peak transmission months.

## Background

Dams and irrigation schemes resulting from water resource development projects (WDP) transform ecosystems and can exacerbate a number of potential breeding sites for mosquito vectors due to a higher availability of water bodies and microclimate modifications. However, there is conflicting evidence on the potential role of WDP in the malaria burden in Africa based on variations in endemicity, seasonality and the type of water management and drainage systems [1–4]. In stable transmission conditions of West Africa, malaria incidence or prevalence remains comparable between irrigated rice cultivation areas and non-irrigated areas [5, 6]. However, WDP may create favourable conditions for malaria transmission in the dry season causing a shift from seasonal to perennial transmission in other areas [7]. For instance, a shift in malaria transmission pattern has been observed in villages near the Manantali dam reservoir in Mali, which were previously characterized by seasonal transmission [2]. Similarly, for irrigated rice cultivation areas of Office du Niger, located in Sahelian Mali, the incidence of malaria fever has been shown to be relatively constant over time, compared with remote, non-irrigated areas where malaria peaks during the rainy season (July to October). Interestingly, malaria incidence has been measured to be two-fold lower in rice cultivation areas compared to non-irrigated areas [5]. This phenomenon, the so-called ‘paddies paradox’, has been explained by improved socio-economic status, effective vector control programmes or changes in health-seeking behaviour in the irrigated areas [7–9].

**Fig. 1:**
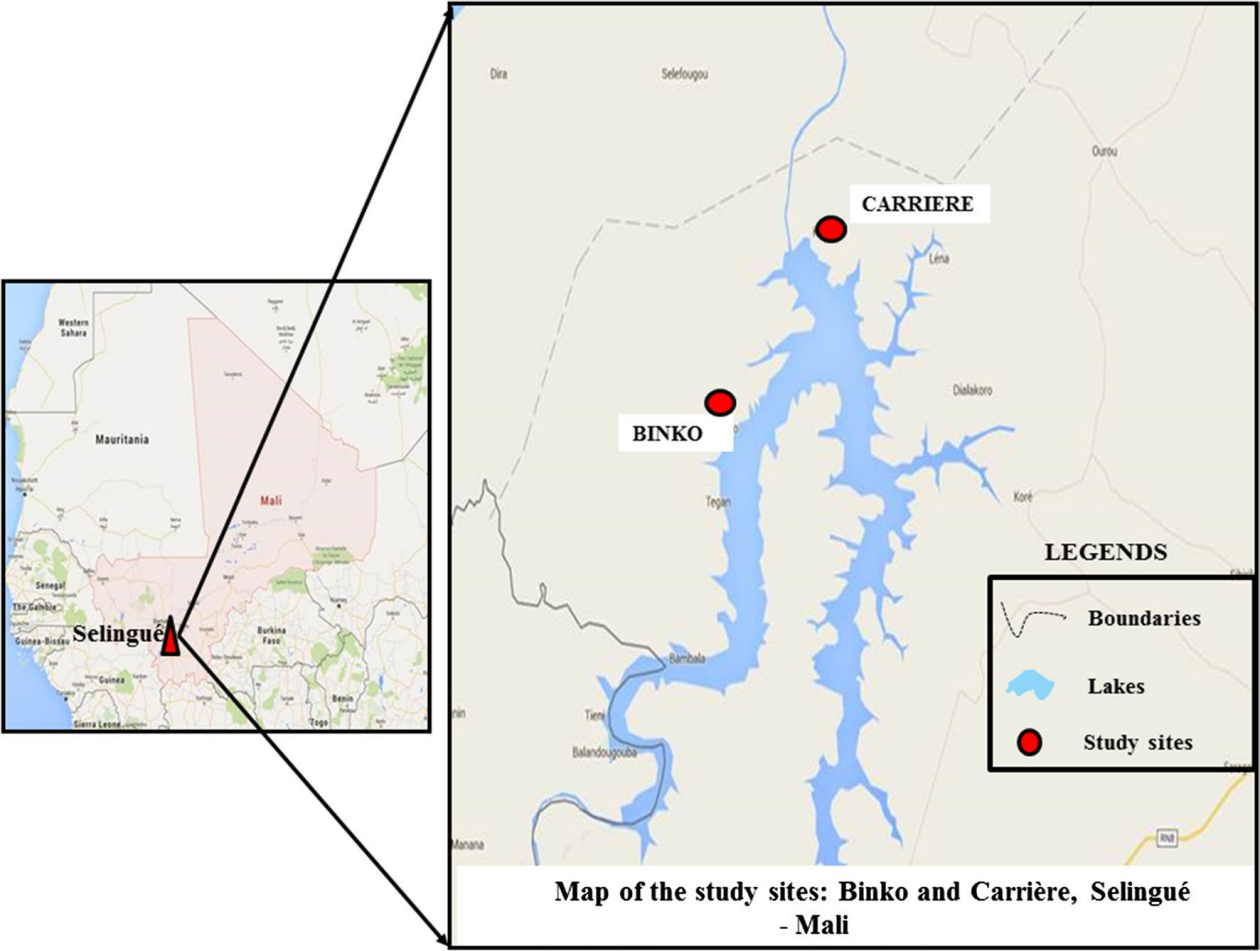
Maps of the study sites, Carrière and Binko. Geographic coordinates of study sites were projected on *Google Earth* image of June 2013 to illustrate the location of each site according to their position by the lake

In Mali, the agro-hydropower dam of Sélingué was built in 1982 and represents the most important centre of energy production in Mali, of approximately 200 million kW-h a year. The presence of an artificial lake introduces a change in the environmental setting of Sélingué with villages located in a dry area such as Carrière, and others in more humid areas with permanent presence of water pools due to irrigation, such as Binko (Fig. 1). The population in villages around Sélingué has two main activities: agriculture in Binko surrounding the irrigated field, while in Carrière fishing is more frequent. The dominant ethnic group is Malinke in the agricultural areas, while Bozo are dominant in the fishing areas. This latter group also presents a high rate of movement within other fishing regions of Mali, such as Mopti and Ségou.

Prior to the impoundment of the dam, the prevalence of asymptomatic malaria parasitaemia followed the classic age pattern of malaria transmission in Mali, increasing from 47.2 % among children <5 years old to 58.1 % among older children. In contrast, the burden of malaria illness was in children under 5 years due to lack of acquired immunity (premunition), while older children remained mostly asymptomatic with parasitaemia [10]. Published data on the epidemiology of malaria in Selingué following the establishment of the agro-hydropower dam and introduction of irrigation schemes are unavailable. Consequently, malaria control interventions up until the time of this study have been focused primarily on distribution of long-lasting insecticide-treated nets (LLINs) and access to free artemisinin-based combination therapy (ACT) for children under 5 years and pregnant women. The aim of this study was to provide baseline up-to-date information on the epidemiology of malaria in relation to the irrigation schemes of the agro-hydroelectric power dam. Understanding the seasonal pattern and age distribution of malaria incidence provides an evidence-basis for improved planning of control interventions, such as the optimal timing of indoor residual spraying (IRS) and distribution of seasonal malaria chemoprophylaxis (SMC).

## Methods

### Study area and population

Sélingué is located in the Guinean Sudan Savanna areas of Mali, 130 km from the capital Bamako. The climate features a dry season from November to May and a wet season from June to October. The Sélingué Dam is a hydro-electric and irrigation dam brought into service in 1982 and located in the Sikasso Region, on the Sankarani River, one of the affluents of the Niger River. The retaining basin of the dam forms the artificial Lake Sélingué. The average rainfall over the basin is 1500 mm per year, although the gradient is significant between the more humid south and the drier northern parts of the basin. When full, the lake stores 2.2 km^3^ of water and has an area of 409 km^2^. From June (at the beginning of the rainy season), the water fills the dam, which remains high until January. Then, under the combined effects of evaporation and water output for power production, the water level decreases slowly. Lake Sélingué rises and falls in the rainy and dry seasons allowing agriculture on the irrigated perimetre, primarily rice cultivation and vegetable production, managed by the Office of Rural Development of Selingué. Fishing and aquaculture is sustained on the lake when water levels allow.

The Sélingué Health District is one of the sentinel sites for malaria surveillance maintained by the National Malaria Control Programme (NMPC). It is the smallest health district in Mali covering seven health zones composed of seven to ten villages each. Each health zone has a community health centre run by a nurse providing basic primary care and referring severe cases to the district health centre located at Selingué. This study was conducted in the villages of Binko and Carrière, each hosting a community health centre. The distance from each village to the referral district hospital is 3 km. The two villages have different ecology features. Binko is located 5 km northwest of Lake Sélingué and 2–3 km from the irrigated perimeters where rice is cultivated perennially. The population size estimated from household census is approximately 4608 inhabitants of which 19 % are under 5 years of age and 17 % between 5 and 9 years old. The inhabitants are mostly sedentary farmers involved in rice cultivation and vegetable production. The ethnic distribution is dominated by the Malinke (80 %) followed by Bambara and Bozo at 5 and 4 %, respectively. The remaining 11 % is comprised of Fulani, Songhoi, Sarakolé and Senoufo.

Carrière is located at the northeast edge of the lake in a relatively dry area where ground water pools depend on rainfall. The population size is 2916 inhabitants of which 20 % are under 5 years and 16 % between 5 and 9 years old. This population is primarily formed of Bozo fishermen who migrated from the central Niger Delta (Mopti and Ségou regions). The Bozo represent around 67 % of the population while Bambara and Soninkés represent 4 % each. The remaining 24 % of ethnic groups are described as follows: 3.8 % Sarakolé, 3.5 % Songhoi. Other groups, such as Senoufo, Minianka and Fulani, represented about 8 %. The village is characterized by transient migration as the Bozo fishermen move north for 3–4 months (February–May) each year, when the water level in the lake is low and unfavourable for fishing.

### Data collection

#### Sampling for cross-sectional surveys and assembling of the cohort study

Initially a house-to-house census was carried out in April 2010 to characterize the study population. A total of 1665 and 678 children under 10 years of age were enumerated in Binko and Carrière, respectively. Based on a conservative prevalence of malaria parasitaemia of 40 %, a precision of 5 %, and 95 % confidence interval, the minimum sample size required for a cross-sectional survey was 223 for Binko and 196 for Carrière. Using random selection, a total of 80 households were selected from a list of 323 in Binko (25.0 %) and 61 households out of 165 in Carrière (38 %). Households selected were also participating in the first cross-sectional study. All children aged 6 months to 9 years of age from the selected households were enrolled in the cohort study after informed consent. Individual interviews were carried out with the mother or guardian of enrolled children to obtain information on bed net ownership and usage as well as demographic and socio-economic status according to the Malian national poverty line [11].

#### Cross-sectional surveys of malaria parasitaemia and anaemia

In each village two cross-sectional malariometric surveys were carried out among children between 6 months to 9 years of age. The first survey was conducted in October 2010 (end of the rainy season) corresponding to the peak of malaria transmission season and the second survey in July 2011, 1 month after the beginning of the rainy season. Each survey was carried out at the health center where parents or guardians were invited to bring their children irrespective of health condition. The participation was voluntary and withdrawal was possible throughout the study. The consent form was written in French, but was explained in the local language to participants by the investigators and a literate village representative.

All children enrolled were clinically examined and axillary temperature measured with an electronic thermometer. Finger prick was performed on all children for on-site diagnosis of malaria by rapid diagnostic test (RDT), detecting histidine-rich protein 2-HRP2: Paracheck^®^Pf by Orchid biomedical Systems, Goa, India). Haemoglobin levels were determined using a Hemocue^®^ 301 reader. RDT-positive children with mild malaria were treated with artemether–lumefantrine (AL) according to the national malaria policy, while children with severe malaria received AL upon transferral to the Sélingué district health centre. Microscopy reading of thick blood smears was performed with ocular 10, objective 100 and the species and numbers of malaria parasites were counted per 300 leukocytes. Quality control of microscopy readings was complete on 10 % of randomly selected slides per site.

#### Cohort study for active and passive case detection of malaria

Children enrolled in the cohort study were followed from 1 November, 2010 to 31 October, 2011 by a team composed of one medical candidate and two local guides at each community health centre. Passive detection of cases was defined as parents of enrolled children that seek treatment at the health centre for any symptoms of illness. Active case detection was done on a weekly basis by performing a daily visit to random households to ask for any sick child in the cohort. By the end of a week all households with enrolled participants were visited by the team. Parents of children seeking treatment or seen at home with children with fever were interviewed on the history of illness and the children were examined clinically. Blood smear slides were read retrospectively at the Malaria Research and Training Centre in Bamako, so that the treatment decision was based on the result from RDT on field.

Malaria parasitaemia was defined as malaria infection or parasite carriage. Symptomatic cases in this study were all patients presenting with fever plus malaria infection, while asymptomatic cases were participants with malaria infections without any symptoms at the time of diagnosis.

### Statistical analysis

#### Prevalence of parasitaemia and anaemia

Data were analysed using SPSS version 14.0 for Windows. Descriptive statistics were used to summarize demographic and malaria indicator data. The seasonal variation of fever, asymptomatic and symptomatic malaria (defined as microscopy positive with or without fever, axillary temperature greater than 37.5 °C, respectively) by village and by survey was estimated. Parasite density was stratified for a malaria-positive patient to highlight the level of *Plasmodium falciparum* carriage across age groups. X^2^-test was used to test for differences in age-specific prevalence and between villages. The odds ratio was used to measure the differences in risk among age groups, across villages and between seasons.

#### Incidence of malaria

The monthly cumulative incidences of malaria by health centre were calculated by dividing the number of malaria cases (defined as fever plus positive blood smear) recorded during the period by the total population per month to minimize the effect of short-term migration in the cohort. Because of the difficulty to perform slide reading in the field, treatment of cases was based on RDT results by national policies for malaria treatment at community level. Two or more consecutive malaria episodes occurring within 3 weeks of the first episode were considered recrudescent infections and treated as a single episode.

#### Risk factor analysis

A logistic regression was performed to identify malaria risk factors. The reference group for comparison was the one for children between 5–9 years of age. The risk of households presenting at least one child with malaria during the follow-up period was compared according to household socio-economic status. The socio-economic score was calculated based on the following variables: monthly self-estimated income (above US$150), type of house, type of bedding, presence of in-house electricity, running water, toilet, and/or shower, ownership of a car, motorcycle and/or bicycle.

## Ethical approval

The study was approved by the institutional review board of the Faculty of Medicine and Pharmacy and Dentistry, in Bamako, Mali.

## Results

### Prevalence of parasitaemia and anaemia

In Table 1 the results of malaria indicators from the two cross-sectional survey are presented. The prevalence of parasitaemia based on microscopy, whether asymptomatic or symptomatic, and anaemia, all display a clear seasonal pattern with monthly peaks in August and October (Table 1). The seasonal variation is markedly higher in Carrière compared to Binko. A significant difference in malaria parasitaemia was observed between the two villages at the end of the transmission season (October 2010) with a prevalence of 29.8 % in Binko and 52.0 % in Carrière (p < 0.001). However, there was no significant difference between the same villages at the beginning of the transmission season (July 2011) with a prevalence of 23.8 % in Binko and 18.9 % in Carrière (p = 0.15).

**Table 1:** Malaria indicators identified in cross-sectional surveys (October 2010 and July 2011) in the villages of Binko and Carrière, Selingué Health District, Mali

Malaria indicators	October			July		
	Binko	Carrière	p	Binko	Carrière	p
	n = 255	n = 221		n = 473	n = 196	
Prevalence of parasitemia, n (%)	76 (29.8)	107 (52)	<0.01	113 (23.8)	35 (18.9)	0.15
Prevalence of symptomatic malaria, n (%)	7 (9.2)	17 (15.9)	<0.01	5 (4.4)	5 (4.3)	0.14
Parasite species, n (%)						
*P. falciparum*	72 (94.7)	107 (94.0)	0.63	108 (95.6)	33 (89.2)	0.15
*P. falciparum* + *P. malariae*	4 (5.3)	8 (6.0)		5 (4.4)	4 (10.8)	
*P. falciparum* density (parasites/µl) n (%)						
1 to 1000	44 (57.9)	50 (46.7)	<0.01	108 (95.6)	30 (85.7)	0.01
1001 to 5000	20 (26.3)	31(29.0)	0.84	5 (4.4)	4 (11.4)	0.12
>5000	12 (15.8)	26 (24.3)	0.16	0 (0.0)	1 (2.9)	0.24
*P. falciparum* geometric mean per µl (IQR)	50 (24–77)	35 (14–37)	0.2	108 (72–117)	271 (81–362)	0.01
Gametocyte carriers (%)	7.8	6.8	0.89	1.9	6.7	<0.01
Anemia, n (%)						
Mild anemia (7.5 g/dl < hb < 9.5)	47 (18.4)	60 (27.2)	0.02	77 (16.2)	32 (16.6)	0.98
Severe anemia (hb < 7.5 g/dl)	25 (9.8)	16 (7.2)	0.32	22 (4.7)	7 (3.6)	0.53
Total prevalence of anemia	72 (28.2)	76 (34.4)	0.14	99 (20.9)	39 (20.2)	0.99

The prevalence of symptomatic malaria was also higher in October for both villages, showing a statistiaclly significant difference between Binko (9.2 %) and Carrière (15.9 %), p < 0.01. *Plasmodium falciparum* represented more than 89 % of the *Plasmodium*-positive infections in both surveys. When stratifying the density of *P. falciparum* infections among children with positive parasitaemia, the majority of children were infected with <1000 parasites/µl in both villages. The difference between villages was significant only for the range 1–1000 parasites/µl, (p < 0.001 at the end of the transmission season and p = 0.01 at the beginning of the transmission season). The geometric mean of parasites/µl (95 % CI) was statistically different between the two villages at the beginning of the transmission season with 271 (81–362) for Carrière and 108 (72–117) for Binko (p = 0.01).

Gametocytes carriage rate shows a significant difference between the two sites in July with, respectively, 1.9 % in Binko and 6.7 % in Carrière (p < 0.01). The overall prevalence of anaemia among children was 28.2 % in Binko and 34.4 % in Carrière in October (p = 0.14) compared to 20.9 % in Binko and 20.2 % in Carrière in July (p = 0.99). The differences between surveys for the same village were not statistically significant (p > 0.05). When stratifying by mild and severe anaemia, Carrière presented a higher prevalence of mild anaemia (27.2 %) compared to Binko (18.4 %), at the end of the transmission season (p = 0.02).

Table 2 shows the Mantel–Haenszel Odds ratio (OR) by age group for malaria infection. For both seasons, the prevalence of malaria infection was significantly higher among children between 5–9 years compared to children below the age of 5 with an OR = 2.3 (1.6–3.4) in October and 3.7 (2.6–5.5) in July.

**Table 2:** Malaria age-specific prevalence per survey in Binko and Carrière

Age groups (years)	October		Mantel–Haenszel OR 95 CI	July		Mantel–Haenszel OR 95 CI
	Binko	Carrière		Binko	Carrière	
	n (%)	n (%)		n (%)	n (%)	
<5	28 (23.7)	39 (37.5)	2.3 (1.6–3.4)	41 (14.4)	14 (37.6)	3.7 (2.6–5.5)
5 and +	52 (32.7)	73 (62.4)		71 (10.4)	21 (34.4)	

**Fig. 2:**
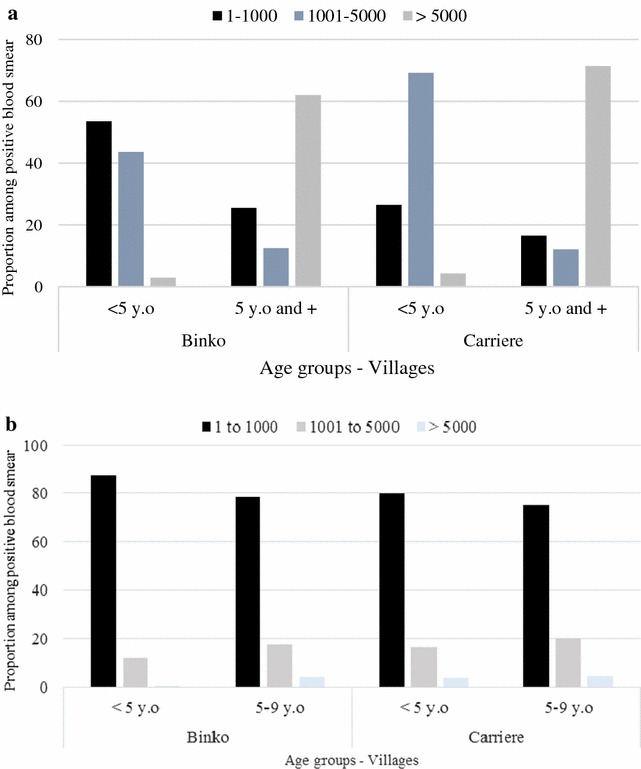
Malaria *Plasmodium falciparum* density/µl among age groups in October 2010 **a** and July 2011 **b** for Binko and Carrière. Number *P. falciparum*/µl of blood was counted in infected children classified by age group and per village. *Black* represent density range from 1–1000 *P. falciparum*/µl *Blue* lighter represent density range from 1001–5000 *P. falciparum*/µl and *White darker* represent density range from 5000+ *P. falciparum*/µl. *Blue bars* represent *Binko* and *red bars* represent Carrière

The parasite densities varied significantly between season and age groups. During the June survey, the proportion of high parasitaemia among infected children (density greater or equal to 5000 *P. falciparum* parasites/µl) was observed among children aged 1–4 years in Binko and children 5 years old and over in Carrière with respectively 8.3 and 9.5 %. Whereas during the October survey, respectively 43.6 and 69.2 % of infected children aged from 1–4 years in Binko and Carrière ranged from 1001–5000 *P. falciparum* parasites/µl while among infected children 5 and more years old, 62 % in Binko and 71.4 % in Carrière had a parasitaemia equal or greater than 5000 *P. falciparum* parasites/µl (Fig. 2a, b).

### Demographic characteristics of the cohort study population

The cohort study was initiated on 1 November, 2010 and continued throughout October 2011. A total of 640 children (340 in Binko and 300 in Carrière) from 6 months to 9 years of age were enrolled. The study participants lived within 80 households in Binko (one-quarter of village total) and 61 households in Carrière (one-third of village total). In Binko, 87.1 % (296/340) of the participants were successfully followed during the 1-year period compared to 84.3 % (253/300) in Carrière. The majority of the subjects lost-to-follow-up had their households moved to another village/town for more than 3 months. The baseline characteristics of the study population are presented in Table 3. The mean age of the participants was 3.9 ± 2.1 years in Binko and 3.6 ± 2.2 years in Carrière (p = 0.05) with males representing 57.4 % in Binko and 47.3 % in Carrière (p = 0.03). In Binko children under 1 year old represented only 5.7 % as opposed to 14.2 % in Carrière. More children between 5 and 9 years of age were included in Binko (32.5 %) compared to Carrière (26.5 %). In both villages, more than half of the parents of enrolled children had never attended school. In Binko about 3.7 % (n = 11) have gone to school for more than 12 years compared to 0.8 % (n = 2) in Carrière. Overall, the difference in parents’ education level between the two villages was not statistically significant (p = 0.14). No significant difference in socio-economic status was observed (p = 0.86) between Binko and Carrière where, respectively, 72.5 and 70.4 % of the households were below the national rural poverty line.

**Table 3:** Demographic data for participants of cohort study in villages Binko and Carrière, Selingué Health District, Mali (November 2010–October 2011)

	Binko (n = 296)	Carrière (n = 253)	p value
Mean age in year (SD)	3.9 (2.1)	3.6 (2.2)	0.05
Sex (male %)	57.4	47.4	0.03
Age groups in years			
Less than 5 years old	200 (67.5)	186 (73.5)	<0.001
5 to 9 years old	96 (32.5)	67 (26.5)	
Education level of parents/guardians (%) (years)			
None	152 (51.4)	140 (55.3)	0.14
<9	96 (32.4)	81 (32.0)	
9–12	37 (12.5)	30 (11.9)	
More than 12	11 (3.7)	2 (0.8)	
Household socio economic status (%)			
Below rural poverty line	58 (72.5)	43 (70.4)	0.86
Above rural poverty line	22 (27.5)	18 (29.6)	

### Incidence of acute malaria and anaemia

In Binko, 151 children (52.3 %) experienced a total of 195 episodes of acute clinical malaria while the remaining 145 children (47.7 %) did not experience any malaria-related symptoms. In Carrière, 134 children (52.9 %) experienced 141 episodes of clinical malaria and 119 children (47.1 %) did not. Table 4 presents data on the incidence of malaria and anaemia during the one-year follow-up period. The cumulative incidence of mild malaria was 55.1 % in Binko and 42.7 % in Carrière (p = 0.07; for severe malaria, the cumulative incidence was 10.6 % in Binko and 13.0 % in Carrière (p = 0.25).

**Table 4:** Malaria incidence, multiple attack rates and anaemia among children (<9 years) in villages Binko and Carrière, Selingué Health District, Mali

	Binko	Carrière n = 253	p value
	n = 296		
Cumulative malaria incidence, %			
Mild malaria	55.1	42.7	0.07
Severe malaria	10.6	13	0.25
Episodes of acute malaria per child (%)			
0	83 (28.0)	89 (30.1)	0.29
1	102 (34.7)	95 (32.1)	
2–3	40 (13.5)	23 (7.8)	
4–5	1 (0.3)	2 (0.7)	
Identified parasite species in malaria cases, %			
*P. falciparum*	91.9	88.6	0.26
*P. malariae*	1.9	7.5	
*P. falciparum* + *P. malariae*	6.2	3.9	
Geometric mean for *P. falciparum* (IQR)	137 (40–259)	169 (62–315)	0.65
*P. falciparum* density per µl (%)			
1–1000	70.8	68.4	0.7
1001–5000	19.1	12.5	0.24
>5000	10.1	19.1	0.07
Anemia prevalence (%)			
Mild anemia (7.5 g/dl < hb < 11)	45.3	47.8	0.83
Severe anemia (hb < 7.5 g/dl)	15.9	15.4	
Total anemia prevalence	61.2	63.2	

The majority of children, 70.8 % in Binko v*ersus* 68.4 % in Carrière, presented a *P. falciparum* density between 1 and 1000 parasites/µl. in both villages. Only 10.1 % in Binko and 19.1 % in Carrière had infections of >5000 parasites/µl. No significant difference was observed when comparing the distribution of parasite density among infected children between the two villages. The prevalence of anaemia was greater than 60 % in both villages of which, mild anaemia represented 45.3 % in Binko and 47.8 % in Carrière, respectively (p = 0.83).

**Fig. 3:**
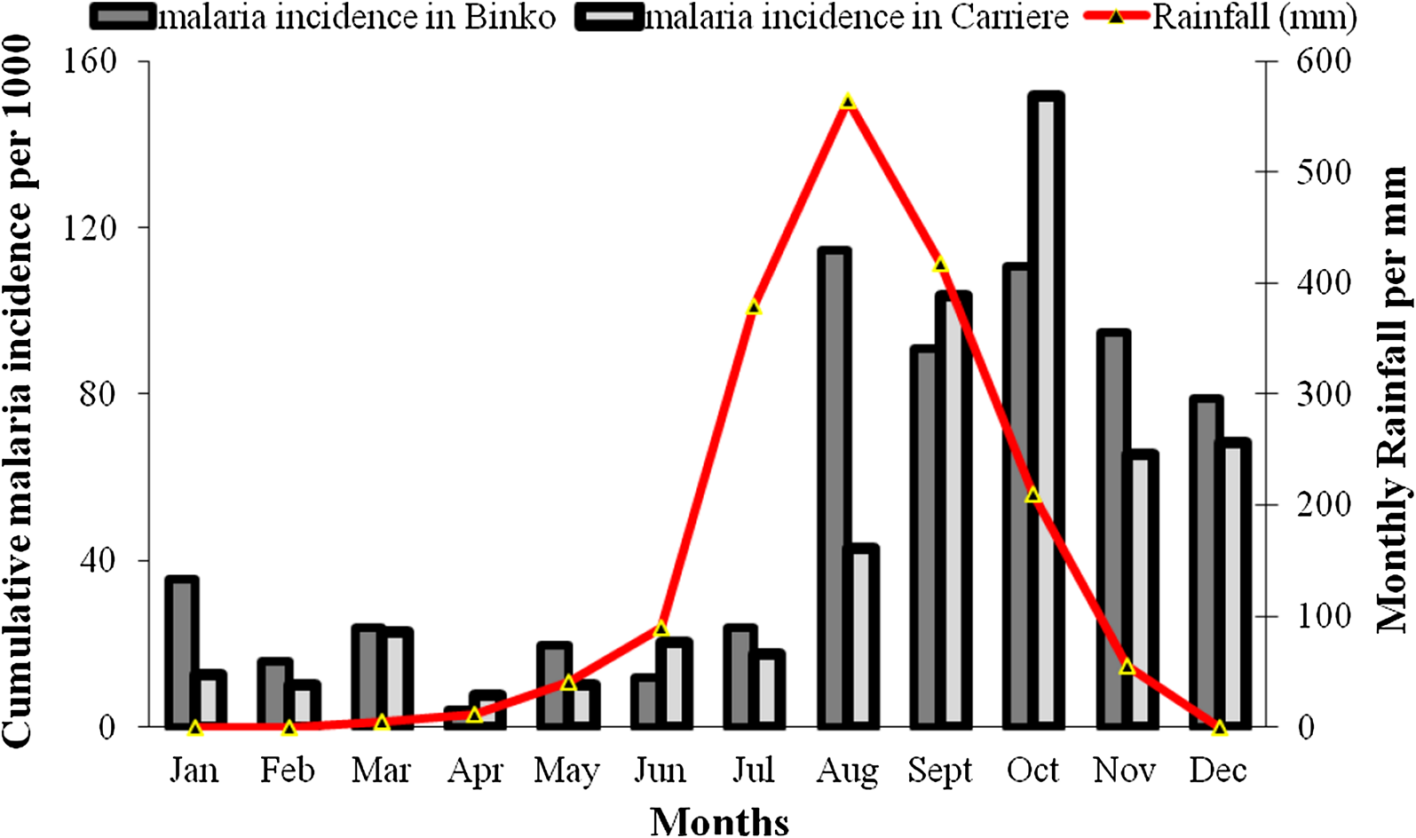
Monthly variation of malaria incidence *versus* rainfall (mm) per site November 2010 to October 2011. Rainfall data were obtained from the local meteorological station during the study and *plot *incidence for each site accordingly. The cumulative incidence was estimated for 1000 children per month

Malaria incidence showed a clear seasonal pattern with 65–70 % of all new malaria cases occurring between August and January, with a peak in October corresponding with the end of the rainy season. The incidence was very low from February to June (five to 40 per 1000) but never reached zero. Malaria incidence was below 50 % during the dry season whereas an increase was observed approximately 1 month after the rainy season start (June) and peaked in October (Fig. 3).

The risk of acute malaria increased with age group in both villages (Table 5). In Binko, age groups 1–4 years and 5–9 years displayed a relative risk of 1.7 (95 % CI 0.7–4.1) and 2.7 (95 % CI 1.2–6.5), respectively, when compared to children under 1 year. In Carrière, the relative risk of malaria infection was 5.8 (95 % CI 1.9–17.5) and 8.5 (95 % CI 2.8–25.7) for the age groups 1–4 and 5–9 years, respectively, as compared to children under 1 year (Table 5).

**Table 5:** Age-specific relative risk (RR) of malaria infection in villages Binko and Carrière, Selingué Health District, Mali

Age groups (years)	Binko (n = 296)			Carrière (n = 256)		
	n	Incidence per 100	RR	n	Incidence per 100	RR
<5	200	19.5	0.4 (0.3–0.4)	189	14.9	0.3 (0.2–0.4)
5+	96	54.2		67	52.5	

### Risk factors for acute malaria

A comparison of malaria disease risk between different age groups across the study population of the cohort study was performed. Several risk factors were examined, including malaria infection at enrolment, age, socio-economic status, and bed net ownership. Only parameters shown to be significant are presented in Table 6.

**Table 6:** Logistic regression of risk factors associated with acute malaria in villages Binko and Carrière, Selingué Health District, Mali

Risk factors	Category	OR	95 % CI	p
Malaria infection at enrolment (Ref: malaria positive)	–	0.1	(0.08–0.2)	<0.01
Age groups (Ref: age 5–9 years)				<0.01
	0–1	0.4	(0.2–0.8)	0.02
	1–2	0.1	(0.3–0.8)	0.01
	2–4	0.9	(0.6–1.6)	0.85
Household socioeconomic status (Ref: above national poverty line)	–	3.2	(2.1–4.8)	<0.01

Children free of malaria infection at the time of enrolment had a significantly lower risk of experiencing clinical malaria during the one-year follow-up period (OR = 0.1; 95 % CI 0.08–0.2; p < 0.01). Age was also a significant risk factor as the odds ratio of clinical malarial was lower for all ages below five. The lower risk was statistically significant for children under 1 year of age (OR = 0.4; 95 % CI 0.2–0.8; p = 0.01) and 1–2 years of age (OR = 0.1; 95 % CI 0.3–0.8) as compared to children over 5 years (p < 0.01). The risk of being positive for malaria was nine-fold higher in children aged 5–9 years compared to children aged up to 2 years in these villages. The socio-ecomic status of the household was highly associated with malaria as households below the national poverty line displayed a 3.2 higher risk of having at least one child with malaria as compared to households above the poverty line (OR = 3.2; 95 % CI 2.1–4.8; p < 0.01).

## Discussion

### Malaria parasitaemia and micro-environmental factors

Malaria epidemiology is known to be dependent on several factors including socio-economic factors, access to health care and the local environment [9]. Prior to the impoundment of the Sélingué Dam in the early 1980s, the prevalence of asymptomatic malaria parasitaemia varied from 47.2 % among children <5 years old to 58.1 % among the 5 and more years old living in the study area during dry season (February–March) [12]. Data suggest that more than 30 years after the dam was created, the prevalence of malaria parasitaemia remains relatively high in the area. The overall prevalence of malaria parasitaemia (indicator of malaria endemicity) varied from 30 % in the village of Binko to 52 % in the village of Carrière at the peak of the transmission period in October 2010. The observed prevalence is consistent with that of the national malaria survey carried out in 2010 showing an overall prevalence of malaria parasitaemia of 62 % in the region of Sikasso as well as other sites in Mali [12, 13]. The difference in prevalence between the two villages is most likely associated with local micro-environmental conditions. Carrière is located at the edge of Lake Sélingué (northeast) in a relatively dry area where ground water pools are replenished by rainfall. From April to June the receding of water from the lake may create multiple smaller pools along the lake that may maintain a low density of malaria vectors, specifically *Anopheles gambiae,* and therefore contribute to sustain malaria transmission during the dry season. In contrast, Binko is located at 3–4 km from the rice fields of the irrigated perimeter. The malaria transmission pattern for this village may be associated with the practices of irrigated rice cultivation, as irrigation schemes have been found to increase the densities of *An. gambiae s.s.* during the early crop stages in the dry season [16, 17]. These observations are consistent with other studies that reported high density of *An. gambiae s.l.* during the dry season in the fishing hamlet Fourda, located in the immediate vicinity of the River Niger compared with the Kéniéroba, a more distant village from the river [14, 15]. These differences in malaria transmission patterns were attributed to variations in micro-environmental conditions between the two areas. A malaria control strategy, including season larval control and environmental management targeting these micro-environmental conditions, may reduce the burden of malaria in the areas. Other confounding factors, such as population mobility not measured in this study, may have played a role as well.

### Age-dependant malaria infection and control practices

The high prevalence of asymptomatic parasitaemia reaching up to 58 % during malaria transmission contrasts with a high coverage of LLINs. Since 2007, the NMCP has organized regularly free distribution of LLINs to households with children under 5 years old (one net for one to two children). In this study, about 93 % of households reported that they owned at least one LLIN and 82 % of children below 10 years old reportedly slept under an LLIN the night prior to the survey. Both ownership and utilization rates are higher in this study compared with the national DHS 2010 (85 % household ownership *versus* 70 % utilization among children under 5 years of age) [12]. The high ownership and utilization rates may be explained by additional free net distribution in the district with support from NGOs, such as BØRNEfonden and Doctors without Borders. As information on net usage was obtained by interview only, the actual rates of net usage could have been overestimated. However, the rate of LLIN usage reaches up to 90 % when analysis was restricted to households owning at least one LLIN.

The risk of clinical malaria was twice as high among children aged 5–9 years compared to children between one and 4 years of age. This constitutes a shift in age groups at high risk for clinical episodes of malaria from children below five to the 5–9 years old living with malaria as generally reported [5, 19, 20]. The observed shift may be related to the malaria control policy, which was focused on children under 5 years and pregnant women at the time of the study. An association between the shifts in malaria affected age groups and LLIN coverage has also been reported elsewhere [21]. It is suggested that the focus on children under 5 years may cause a delay in the acquisition of natural immunity, rendering children at increased risk of acute infection once they have outgrown the age-specific control interventions, at 5 years and older. At the time of the study, LLINs were provided free of charge to children under 5 years and pregnant women. However, in this study the coverage and usage rates of LLINs were relatively higher, presumably because of mosquito nuisance in irrigated areas [5, 24]. The national policy for malaria treatment recommends free anti-malarials, with ACT (AL) as first-line drug, for children under 5 years old and pregnant women only. Mali has adopted universal LLINs coverage since 2011, and control interventions such as free treatment strategy remain focused on children under five while SMC covers only children under 2 years old. These findings suggest the need to expand both strategies to school-age children to cover the peak transmission months in order to meet an important change in the disease burden.

Severe malaria, also described in this study, was mainly dominated by severe anaemia with a cumulative incidence of 15 % in both villages. Anaemia remains a major public health issue and requires further studies to identify the causes of anaemia among children in the region. Several competing causes of anaemia, including schistosomiasis and worm infections, are heavily present [13]. Further studies are needed to characterize the causes of anaemia in the study area.

### Seasonality in malaria parasitaemia and incidence of the disease

Although malaria is present throughout the year in the area, the prevalence of parasitaemia as well as the clinical disease incidence showed a seasonal pattern with a clear peak at the end of the rainy season in October. Other studies conducted in Mali have reported a similar transmission peak at the end of the rainy season (usually in October or November) [20–23]. Unlike these studies, the incidence of malaria remained relatively high two to 3 months after the end of the rainy season (through January). A plausible explanation could be the persistence of some vector breeding sites in the dry season combined with a decrease in personal protection due to a lowering of the general mosquito density. This was mainly observed during the dry season where water is almost absent in the rice fields, reducing mosquito density considerably in the surrounding village [7]. This may support the notion that irrigation projects impact differently on malaria transmission and these projects can be associated with the increase in vector capacities at lower density and its decrease at higher densities [1–7]. However, the bimodal transmission pattern with a secondary peak of malaria illnesses during the dry season as reported in other irrigation areas was not observed in the study area [5, 24]. Moreover, more than 80 % of the malaria cases occurred between August and January while the incidence decreased considerably (below 5 %) from January through June. The two peaks observed by Sissoko et al. [5] were associated with the rainy season from July to October and the double cropping season in March and April during the dry season. The absence of this second peak during the dry season in Sélingué could be explained by the effective water management system in the area compared to Niono irrigated area of Mali [5, 24]. The current water management system in Sélingué may prevent the proliferation of mosquito breeding sites during the dry season, even with the double cropping in the rice fields located near Binko. The seasonality of malaria parasitaemia and incidence of the disease in Sélingué offers opportunities for an integrated malaria control strategies, including SMC and vector control strategies, such as a targeted dry season larval control and environmental management. It also highlights the need for a search of other causes of fevers that may be treated by local health providers as presumptive malaria during the dry season.

### Prevalence of anaemia

Anaemia is prevalent among children in the study area, specifically among the under 5 years old. This study does not find a significant correlation between anaemia and malaria infection. The finding calls for probably the presence of other causes of anaemia. Other parasitic diseases, mainly schistosomiasis, are highly endemic in the area. The prevalence of *Schistosoma haematobium* in Sélingué is up to 85.9 % among schoolchildren with 43.8 % representing heavy infection. The transmission of *Schistosoma mansoni* is confined to the Sélingué Dam area with a prevalence of 12.5 % [18]. This study did not determine specific causes for anaemia in Sélingué, which could be a research perspective in the area.

## Conclusion

Malaria incidence remains highly seasonal in Sélingué but prolonged over 2–3 months after the rainy season, presumably associated with irrigation related to the agro-hydropower. No secondary peak during the dry season in relation to the irrigation scheme was observed. Since malaria prevention strategies and case management target specific vulnerable groups (children under 5 years of age and pregnant women), this study shows that the proportion of children over five carrying malaria parasites in these communities is much higher at any season compared to the younger ones. If Mali has adopted universal LLINs coverage since 2011, control interventions such as free treatment strategy remains focused on children under five while SMC covers only children under 2 years old. The findings call for expanding both SMC and free ACT to school-age children to cover the peak transmission months in order to meet an important change in the disease burden.

## Abbreviations

ACT: artemisinin-based combination therapy
AL: artemether-lumefantrine
CI: confidence interval
DHS: demographic and health survey
IRS: insecticide residual spraying
LLIN: long-lasting insecticide-treated net
MRTC: Malaria Research and Training Centre
NMPC: National Malaria Control Programme
NGOs: non-governmental organizations
OR: odds ratio
RDT: rapid diagnostic test
RR: relative risk
SMC: seasonal malaria chemotherapy
USTTB: University of Sciences, Techniques and Technologies of Bamako
WDP: Water Resource Development Projects
